# Pooled week 48 safety and efficacy results from the ECHO and THRIVE phase III trials comparing TMC278 vs EFV in treatment-naïve, HIV-1-infected patients

**DOI:** 10.1186/1758-2652-13-S4-O48

**Published:** 2010-11-08

**Authors:** C Cohen, JM Molina, P Cahn, B Clotet, J Fourie, B Grinsztejn, W Hao, M Johnson, K Supparatpinyo, HM Crauwels, L Rimsky, S Vanveggel, P Williams, K Boven

**Affiliations:** 1 Community Research Initiative of New England, Boston, MA, USA; 2 Saint-Louis Hospital and University of Paris, Department of Infectious Diseases, Paris, France; 3 Hospital Juan A Fernández and Fundación Huesped, Buenos Aires, Argentina; 4 Hospital Universitari Germans Trias i Pujol and irsiCaixa Foundation, UAB, Barcelona, Spain; 5 Dr J Fourie Medical Centre, Dundee, KwaZulu Natal, South Africa; 6 Instituto de Pesquisa Clínica Evandro Chagas-Fiocruz, Rio de Janeiro, Brazil; 7 Beijing You’an Hospital, Beijing, China; 8 Royal Free Hospital, London, UK; 9 Chiang Mai University, Section of Infectious Disease, Chiang Mai, Thailand; 10 Tibotec BVBA, Beerse, Belgium; 11 Tibotec Inc, Titusville, NJ, USA

## Introduction

Pooled 48-week primary analysis results of two double-blind, randomised, TMC278 Phase III trials, ECHO (TMC278-C209, NCT00540449) and THRIVE (TMC278-C215, NCT00543725), are presented.

## Methods

Treatment-naïve adult patients (N=1368) received (1:1) TMC278 25mg qd or EFV 600mg qd, plus TDF/FTC (ECHO), or TDF/FTC, AZT/3TC or ABC/3TC (THRIVE). The primary objective was to demonstrate non-inferiority (12% margin) of TMC278 to EFV in confirmed virologic response (viral load [VL] <50 copies/mL ITT-TLOVR algorithm) at Week 48.

## Results

**Figure 1:**
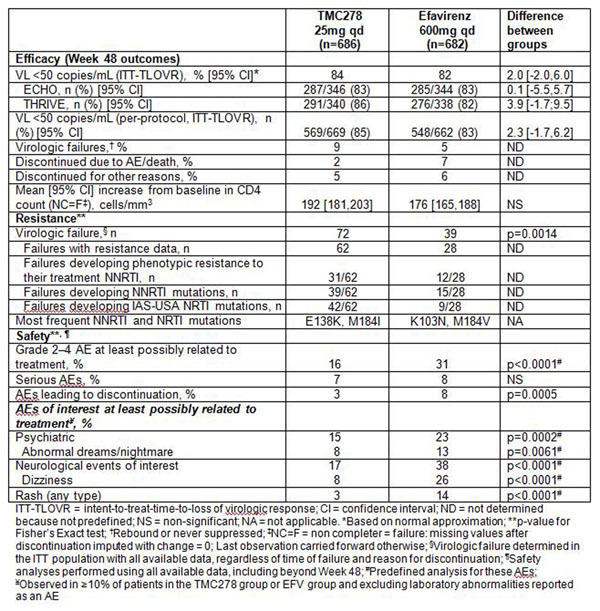


Overall virologic response rates at Week 48 were high (Figure 1).TMC278 showed non-inferior efficacy versus EFV. The impact of adherence, in addition to other factors, such as baseline viral load and exposure, on virologic response will be presented. Incidences of the following tolerability measures were significantly lower in the TMC278 group than in the EFV group: adverse events (AEs) leading to discontinuation (3% vs. 8%, respectively; p=0.0005), grade 2-4 AEs at least possibly related to treatment (16% vs. 31%; p<0.0001), rash (3% vs 14%; p<0.0001), dizziness (8% vs. 26%; p<0.0001), abnormal dreams/nightmare (8% vs. 13%; p=0.0061), and grade 3/4 laboratory abnormalities for lipids (p≤0.001).

## Conclusions

At Week 48, TMC278 demonstrated a high virologic response rate (≥83%) and non-inferior efficacy versus EFV when administered with NRTIs in both Phase III trials. The virologic failure rate was significantly higher with TMC278, while the incidences of AEs leading to discontinuation were significantly lower with TMC278. Grade 2-4 AEs at least possibly related to treatment were half as frequent with TMC278 compared with EFV. In addition, incidences of dizziness, abnormal dreams/nightmare and rash were significantly lower for TMC278, and TMC278 had significantly fewer grade 3/4 lipid abnormalities than EFV.

